# Radial Flow Assay Using Gold Nanoparticles and Rolling Circle Amplification to Detect Mercuric Ions

**DOI:** 10.3390/nano8020081

**Published:** 2018-02-01

**Authors:** Tai-Yong Kim, Min-Cheol Lim, Min-Ah Woo, Bong-Hyun Jun

**Affiliations:** 1 Food Safety Research Group, Korea Food Research Institute (KFRI), Jeollabuk-do 565-851, Korea; kim.tai-yong@kfri.re.kr (T.-Y.K.); mclim@kfri.re.kr (M.-C.L.); 2 Department of Bioscience and Biotechnology, Konkuk University, Seoul 143-701, Korea

**Keywords:** mercuric ion, thymine-Hg^2+^-thymine, gold nanoparticle, rolling circle amplification, colorimetric detection, radial flow assay

## Abstract

A novel colorimetric assay employing oligonucleotide-conjugated gold nanoparticle (AuNP probes) and rolling circle amplification (RCA) was developed for simple detection of mercuric ions (Hg^2+^). The thymine-Hg^2+^-thymine (T-Hg^2+^-T) coordination chemistry makes our detection system selective for Hg^2+^. In the presence of Hg^2+^, the thymine 12-mer oligonucleotide is unable to act as a primer for RCA due to the formation of T-Hg^2+^-T before the RCA reaction. However, in the absence of Hg^2+^, DNA coils as RCA products are generated during the RCA reaction, and is further labeled with AuNP probes. Colorimetric signals that depend on the amount of DNA coil-AuNP probe complexes were generated by drop-drying the reaction solution on nitrocellulose-based paper. As the reaction solution spread radially because of capillary action, the complexes formed a concentric red spot on the paper. The colorimetric signals of the red spots were rapidly measured with a portable spectrophotometer and determined as the Δ*E* value, which indicates the calculated color intensity. Our assay displays great linearity (detection limit: 22.4 nM), precision, and reproducibility, thus demonstrating its utility for Hg^2+^ quantification in real samples. We suggest that our simple, portable, and cost-effective method could be used for on-site Hg^2+^ detections.

## 1. Introduction

Mercury is one of the most toxic heavy metals, representing a severe threat to the global environment and human health [1]. In particular, Hg^2+^ is one of the most prevalent mercuric pollutants of environmental water [2]. Via the biosynthetic pathways of microorganisms, Hg^2+^ is converted into methyl mercury, which can be easily absorbed by bacteria, plankton, and fishes, and it causes serious damage to the human brain, nervous system, kidneys, and endocrine system [3,4]. The most common methods currently used for mercury detection include inductively coupled plasma–mass spectrometry (ICP-MS) [5], atomic absorption spectroscopy (AAS) [6], and gas chromatography (GC) [7]. Although these methods can detect a wide range of metal ions with high sensitivity and selectivity, they require expensive and sophisticated equipment, trained personnel, and time-consuming and labor-intensive procedures. To overcome these drawbacks, a variety of sensor approaches using nanomaterials for fluorescent detection of Hg^2+^ [8,9,10,11] have emerged due to its capabilities for sensitivity, selectivity, reproducibility, and rapid real-time monitoring [12]. However, they still suffer from expensive and complicated functionalization of the nanomaterials and require sophisticated analytical instruments for readouts (fluorescence spectrophotometer). Therefore, it is necessary to develop a simple and convenient Hg^2+^ detection method to overcome these limitations.

Colorimetric sensors using gold nanoparticles (AuNPs) are attractive because they do not require complex analytical equipment and can be easily interpreted with the naked eye [13,14]. A solution of well-dispersed AuNPs gives a red color, but, when AuNPs are aggregated, they have a violet or blue color. The distance-dependent color change of AuNPs is associated with inter-particle plasmon coupling, leading to a significant shift in the absorption spectrum [15,16]. Various methods to detect Hg^2+^ based on the colorimetric changes of AuNPs have been reported, in the most cases involving the use of thymine (T) probes as receptors selective for Hg^2+^ [17,18,19]. It is well known that Hg^2+^ can bind two thymine residues of DNA to form a T-Hg^2+^-T complex [20,21]. This complex, which has a stronger binding affinity than the Watson-Crick DNA duplex, can stabilize DNA duplexes containing T-T mismatched base pairs [22,23]. Using T-T mismatch formation, DNA probe-modified AuNPs have been utilized to selectively recognize Hg^2+^ and directly exhibit a color change that can be observed with the naked eye [24]. Nevertheless, most of the abovementioned methods still depend on bulky instruments for accurate quantitative analysis, making them impractical for on-site measurement.

Recently, paper-based assays (PBAs) have received considerable attention in the area of point-of-care testing (POCT) [25]. Many PBAs adopt formats with lateral flow assays (LFAs), dipstick assays, and microfluidic paper-based analytical devices (µPADs) [26,27,28]. These formats for PBAs have great advantages in terms of their reasonable price, portability, ease of handling, and low sample/reagent consumption [29,30,31]. In many cases, however, pre-treatment of papers such as micro-fabrication or surface modification is needed for making flow patterns or immobilizing probe molecules on the paper. In several studies aiming to develop on-site Hg^2+^ sensors, AuNPs on paper substrates have been employed for colorimetric assays [32,33,34]. Color changes on the paper, reflecting the interaction between AuNPs and Hg^2+^, are simply captured using a mobile camera, and the color change can then be quantified by using image analysis software [33,34]. Compared with other sensing methods, this approach represents a powerful advance in the practical field monitoring of trace Hg^2+^. However, various factors influencing lighting conditions, such as weather, sunlight strength, and the angle at the point of readouts, affect the reproducibility and accuracy of the assay.

Herein, a novel colorimetric sensing system for detection of Hg^2+^ was developed using AuNPs and rolling circle amplification (RCA). In this method, RCA is driven by the hybridization of (T)12 (thymine 12-mer) primers with the circularized oligonucleotide templates, generating concatemeric micron-sized single-stranded DNA (ssDNA) coils under isothermal conditions. However, RCA is inhibited by the formation of binding complexes consisting of (T)12 primer and Hg^2+^, resulting in varying amounts of DNA coils according to the Hg^2+^ concentration. The DNA coils produced by RCA are labeled with AuNP probes for colorimetric sensing, and they are drop-dried on non-pretreated nitrocellulose (NC)-based paper. The variance of color intensity on the paper is measured using a hand-held spectrophotometer, which is not affected by the lighting conditions. The analytical utility of this new strategy was successfully demonstrated by quantifying Hg^2+^ in tap water with high selectivity, sensitivity, precision, and reproducibility.

## 2. Results and Discussion

### 2.1. Design of Radial Flow Assay for Hg^2+^ Detection

**Figure 1:**
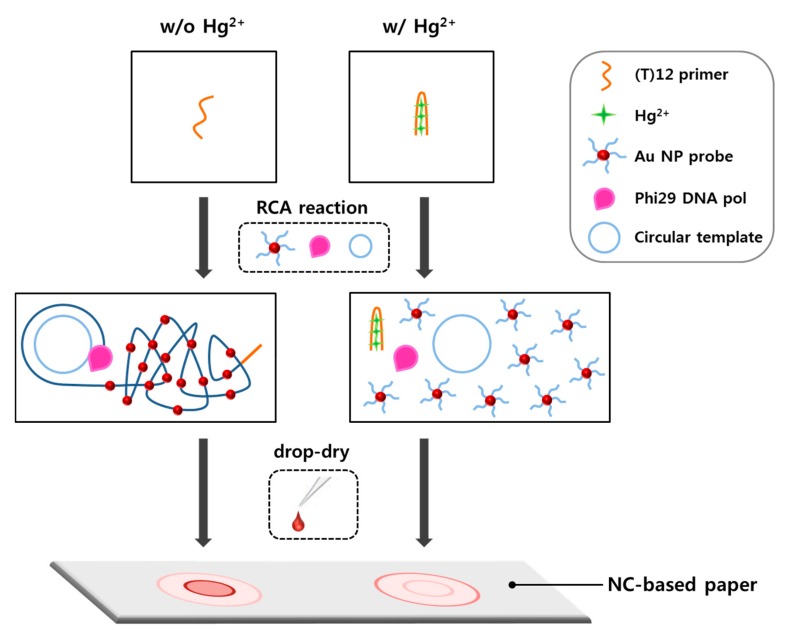
Schematic illustration of radial flow assay for Hg^2+^ detection.

The strategy of the radial flow assay for sensing Hg^2+^ is shown in Figure 1 and is based on the assembly of AuNPs bound with DNA coils, which are generated by RCA. RCA is an isothermal, enzymatic process mediated by certain DNA polymerases in which long ssDNA molecules are synthesized on a short circular ssDNA template using a single DNA primer [35]. RCA is a powerful sensing system because it generates concatemeric micron-sized single-stranded DNA coils that are spatially condensed and detectable as single amplified molecules [36]. In several conventional RCA detection sensors, the RCA reaction is driven by the linking of a padlock probe that is dependent on the presence of a detection target [37,38,39,40]. In contrast, in our detection system, RCA is inhibited by the formation of binding complexes consisting of (T)12 primer and Hg^2+^. In the absence of Hg^2+^, RCA is driven by phi29 DNA polymerase when (T)12 primers hybridize with the circularized oligonucleotide templates. During the RCA reaction, concatemeric micron-sized ssDNA coils are generated under isothermal conditions, and these DNA coils are labeled with AuNP probes. Finally, the reaction solutions are drop-dried on NC-based paper, and the variance of the color intensity of the drop is rapidly and simply measured using a hand-held spectrophotometer within a few seconds. Based on capillary action, the reaction solution travels from the center of the paper toward the periphery. The components of the solution are radially separated in the mobile phase, and spots of different compounds are formed as concentric rings in the stationary phase. In our detection system, the amount of DNA coil-AuNP complex varies with Hg^2+^ concentration, such that the formation of concentric rings and their color intensities on the paper differ according to the concentration of Hg^2+^. DNA coil-AuNP complexes are formed as a spot in the center of the drop, whereas free AuNP probes radially diffuse over a greater distance, consequently forming a concentric ring on the outer edge of the drop. 

### 2.2. RCA Reaction Test Using Circular Template–(T)12 Primer Complexes

**Figure 2:**
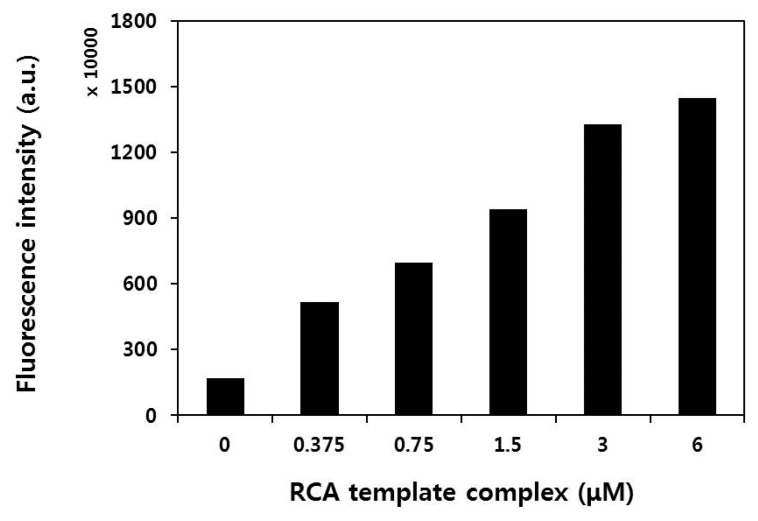
Rolling circle amplification (RCA) reaction test using various concentrations of circular template–(T)12 primer complex.

We successfully produced circular template DNA by ligation of a 54 mer ssDNA, consisting of (A)12 mer (AAAAAAAAAAAA) to be hybridized with the (T)12 primer (Figure S1, Supplementary Materials). An experiment was then performed to confirm DNA amplification through the formation of circular template–(T)12 primer complexes. Different concentrations of circular template–(T)12 primer complexes were tested under the experimental conditions for the RCA reaction, and SYBR Green II was used as a staining reagent to determine the amount of amplified ssDNA coils. Figure 2 shows yields of ssDNA coils according to the amount of circular template–(T)12 primer complex. As the amount of circular template–(T)12 primer complex increased from 0 to 6 μM, the fluorescence intensity of SYBR Green II also increased. This result demonstrates that the prepared circularized oligonucleotides effectively act as a template for RCA and that the amount of ssDNA coil generated by RCA is proportional to the amount of circular template–(T)12 primer complex.

### 2.3. Formation of DNA Coil-AuNP Complexes by RCA Reaction

**Figure 3:**
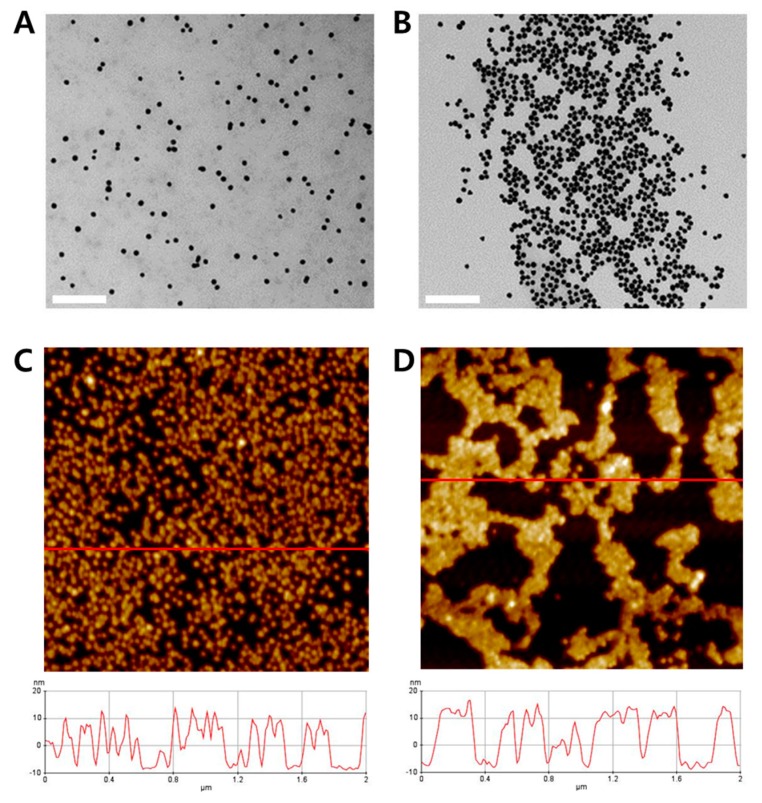
Transmission electron microscopy (TEM) images and atomic force microscopy (AFM) analysis results of: the non-reaction sample with AuNP probes alone (**A**,**C**); and RCA reaction sample with AuNP probes (**B**,**D**), respectively. The length of the inset scale bar in the TEM images is 200 nm. The horizontal red lines of the AFM images indicate the analysis range of height profile, and lower graphs of the AFM images indicate the height profiles of each sample.

To fabricate AuNP probes, the surfaces of streptavidin-conjugated AuNPs (SA-AuNP) were functionalized with biotinylated oligonucleotides. The properties of the AuNP probes were determined by dynamic light scattering (DLS) and zeta potential measurement, as shown in Figure S2A,B, respectively (Supplementary Materials). The morphology of two samples (non-reaction sample with AuNP probes only and RCA reaction sample with AuNP probes) was determined by transmission electron microscopy (TEM). As shown in Figure 3A, AuNP probes in the non-reaction sample were well dispersed in the solution. In contrast, aggregated AuNP probes were observed in the RCA reaction sample owing to the complementary binding of ssDNA coils with the probe DNA on the surface of AuNPs (Figure 3B). We also obtained atomic force microscopy (AFM) images of the two samples and found that the nano-sized wires existed in the RCA reaction sample (Figure 3D). The image and height profile of the sample in Figure 3D showed that RCA generated assemblies of DNA coil-AuNP complexes. Moreover, the thickness of the complexes was 3–5 nm greater than the thickness of the AuNP probes alone, as shown in Figure 3C.

### 2.4. Different Aspects of Concentric Spot Generation on the Paper According to the Presence of Hg^2+^

**Figure 4:**
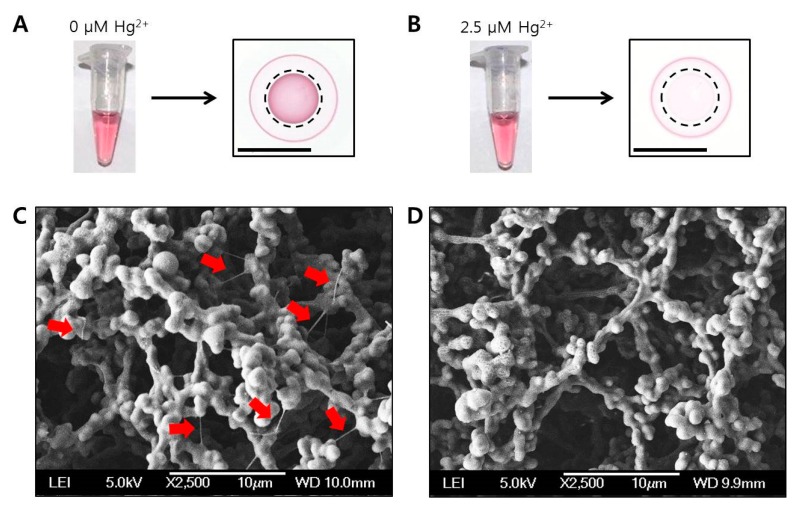
RCA reaction solution in the tube (left) or drop-dried on the paper (right): in the absence of Hg^2+^ (**A**); or in the presence of 2.5 μM Hg^2+^ (**B**). Dotted circles in Figure 4A,B indicate the areas for measuring color intensities using a portable spectrophotometer. The length of the inset scale bars in Figure 4A,B are 10 mm. Scanning electron microscopy (SEM) images of two different papers prepared by drop-drying of the RCA reaction solution: in the absence of Hg^2+^ (**C**); or in the presence of 2.5 μM Hg^2+^ (**D**). Red arrows in the SEM image indicate DNA coil-AuNP complexes. The length of the inset scale bars in Figure 4C,D are 10 μm.

In our detection system, RCA is driven by phi29 DNA polymerase when (T)12 primers hybridize with the circularized oligonucleotide templates in the absence of Hg^2+^. In the normal RCA reaction, concatemeric micron-sized ssDNA coils are generated under isothermal conditions. However, in the presence of Hg^2+^, RCA is inhibited by the formation of binding complexes consisting of (T)12 primer and Hg^2+^. As shown in Figure 4A,B, the color intensities of the two different RCA reaction samples, which were incubated without Hg^2+^ or with 2.5 μM of Hg^2+^, in the tubes were indistinguishable to the naked eye. In contrast, when the two solutions were drop-dried on each paper, different aspects of concentric spot generation were observed, i.e., the red spot in the absence of Hg^2+^ or pale pink spot in the presence of 2.5 μM of Hg^2+^ (marked with dotted circles in Figure 4A,B). To investigate the morphologies of the color spots, each paper was imaged using scanning electron microscopy (SEM). As a result, very thin nano-sized wires (marked with red arrows in Figure 4C) were observed on the red spot generated by drop-drying RCA reaction solution without Hg^2+^. Based on these results, we found that the compounds in the reaction solution were radially separated over the paper in the mobile phase and finally generated concentric red spots in the stationary phase. Therefore, the red color spot on the center of the drop could be generated by the DNA coil-Au NP complexes enmeshed in the porous structure of the paper. In contrast, free AuNP probes diffused over a greater distance than the DNA coil-AuNP complexes, consequently forming a concentric ring on the outer edge of the drop. By using a portable spectrophotometer, the color intensities of the colorimetric spots (marked with dotted circle lines) of Figure 4A,B were simply and measured within a few seconds. The resulting values (Δ*E*) for 0 and 2.5 μM Hg^2+^ samples were 18.6 and 5.4, respectively.

### 2.5. Selectivity of Radial Flow Assay for Hg^2+^

**Figure 5:**
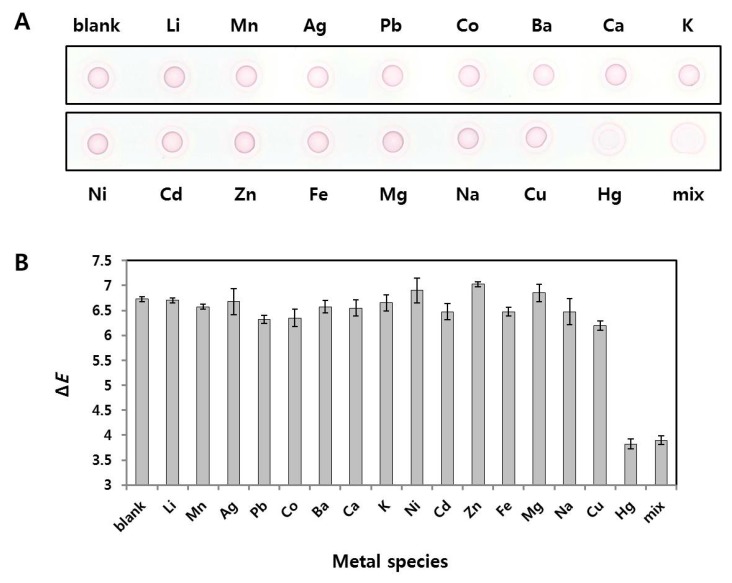
Selectivity of the radial flow assay for Hg^2+^: (**A**) Digital scanned image of red spots formed on NC-based paper. The final concentration of each metal ion was 1 µM; and (**B**) Color intensities (Δ*E*) of the spots as measured by a portable spectrophotometer (*n* = 3).

To confirm the selectivity of the proposed analytical method for Hg^2+^, various metal ions as well as Hg^2+^ were tested in triplicate, and the colorimetric intensities of the spots drop-dried on the paper were compared (Figure 5). The final concentration of each metal ion was 1 µM. Figure 5A shows a digital scanned image of the paper, and Figure 5B shows a bar graph of the color intensities (Δ*E*) as measured by a portable spectrophotometer. The color intensities of the spots on the paper were easily distinguished with the naked eye, especially in the two samples containing Hg^2+^ only and ion mixture containing Hg^2+^, Δ*E* values among other metal ions were also easily determined. The reason for the less intensities in Hg^2+^-containing samples is that the DNA coil-AuNP complex formation was reduced by inhibition of the RCA reaction; thus, free AuNP probes had higher color intensity in the concentric ring on the outer edge of the drop. These results demonstrate that the proposed analytical method is selective for Hg^2+^, and can also be used to determine Hg^2+^ concentrations in unknown samples without interference from other metal ions. The portable spectrophotometer is completely in contact with the paper, such that a constant and reproducible measurement can be possible regardless of variable external lighting conditions. This feature provides an advantage as a sensor for the field detection of Hg^2+^. 

Additionally, the selectivity of the (T)12 primer for Hg^2+^ is shown in Figure S3 (Supplementary Materials). High fluorescence intensities of SYBR Green I were obtained in both samples consisting of Hg^2+^ only and the ion mixture containing 5 μM Hg^2+^ compared with other metal ion samples showing lower intensities under the same conditions. This shows that the single-stranded primer changed to a double-stranded complex due to the coordinated binding of Hg^2+^ between two thymines. Thus, the primer could not further bind to the circular template, inhibiting the RCA reaction.

### 2.6. Quantification of Hg^2+^ Using Radial Flow Assay

**Figure 6:**
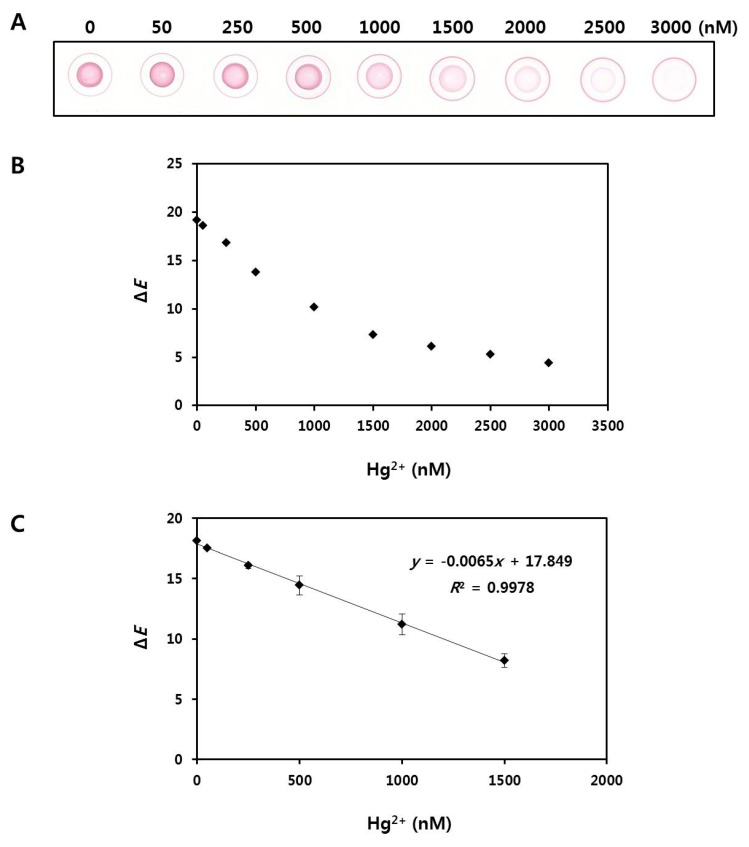
(**A**) Digital scanned image of the paper after drop-drying of nine samples with different Hg^2+^ concentrations (0, 50, 250, 500, 1000, 1500, 2000, 2500, and 3000 nM); (**B**) Color intensities (Δ*E*) of the nine samples measured by a portable spectrophotometer; and (**C**) Linear correlation between Hg^2+^ concentrations (0, 50, 250, 500, 1000 and 1500 nM) and Δ*E* values (*n* = 3).

The correlation between the color intensity of the spots and concentration of Hg^2+^ was evaluated next. Figure 6A shows a digital scanned image of the paper after drop-drying of the reaction solution. A decrease of the colorimetric intensity of the spot in the center of the drop was observed with the naked eye, representing increasing concentrations of Hg^2+^. In contrast, the color intensity of the concentric ring on the outer edge of the drop increased with increasing concentrations of Hg^2+^. The red color spots were measured using a portable spectrophotometer, and the resulting values were used to calculate Δ*E* (Figure 6B). The resulting values for nine samples with varying Hg^2+^ concentrations (0, 50, 250, 500, 1000, 1500, 2000, 2500, and 3000 nM) were 19.2, 18.6, 16.8, 13.8, 10.2, 7.3, 6.1, 5.3, and 4.4, respectively, showing that the Δ*E* value tended to decrease with increasing Hg^2+^ concentration. Additional experiments to obtain a linear standard curve were performed at Hg^2+^ concentrations ranging from 0 to 1500 nM (Figure 6C). Six samples with different Hg^2+^ concentrations (0, 50, 250, 500, 1000, and 1500 nM) were tested in triplicate, and the mean Δ*E* values were 18.1 ± 0.05, 17.5 ± 0, 16.1 ± 0.25, 14.4 ± 0.77, 11.2 ± 0.86, and 8.2 ± 0.57, respectively. A scanned image of the paper of Figure 6C is shown in Figure S4A (Supplementary Materials). The relationship between the resulting values and the Hg^2+^ concentrations was observed to be linear, and the limit of detection (LOD) for Hg^2+^ quantification was calculated to be 21.8 nM at a signal-to-noise ratio of 3 (*n* = 3). The total assay time was within 90 min. The precision and reproducibility of our method for Hg^2+^ quantification were assessed using four different samples spiked with different amounts of Hg^2+^ in distilled water. The coefficient of variation was found to be 0.7–3.2%, which was better than that with ICP-MS (2.7–6.5%). Recovery rates were acceptable with a range of 96–129%, compared with 82.3–117.1% for ICP-MS (Table S1, Supplementary Materials).

### 2.7. Analysis of Hg^2+^ in Tap Water Samples

**Figure 7:**
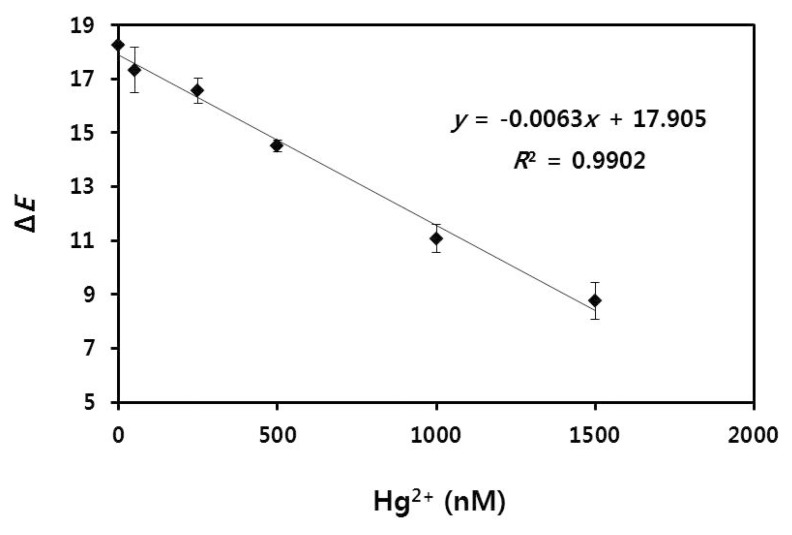
Linear correlation between Hg^2+^ concentrations (0, 50, 250, 500, 1000 and 1500 nM) and Δ*E* values in tap water samples. On the basis of the Δ*E* plot, the detection limit of 22.4 nM was obtained (*n* = 3).

To demonstrate the applicability of our method in environmental samples, different concentrations of Hg^2+^ (0, 50, 250, 500, 1000 and 1500 nM) were spiked into tap water, and the samples were tested using our radial flow assay. Tap water was obtained from the laboratory at our institute and filtered through a cellulose acetate syringe filter with a pore size of 0.2 μm. The analysis results for the colorimetric intensities on the paper are shown in Figure 7 and digital scanned images are shown in Figure S4B. The mean Δ*E* values corresponding to the Hg^2+^ concentrations of 0, 50, 250, 500, 1000 and 1500 nM were 18.27 ± 0.05, 17.33 ± 0.83, 16.57 ± 0.48, 14.5 ± 0.22, 11.07 ± 0.52 and 8.77 ± 0.68, respectively. A linear correlation was found in the relation between Hg^2+^ concentration and Δ*E* value even when Hg^2+^ was present in the real samples. As the limit of detection was calculated to be 22.4 nM at a signal-to-noise ratio of 3 (*n* = 3), we have achieved a detection limit of less than 30 nM as the allowable limit for mercuric ions in drinking water as indicated by the World Health Organization (WHO) [41]. This LOD value is also lower than that of other currently developed paper-based methods that detect mercuric ions in water source based on the color change from red to blue induced by aggregation of AuNPs [33].

The coefficient of variations (CVs) of the within- and between-assays were evaluated using three tap water samples containing different concentrations of Hg^2+^. As shown in Table 1, the reproducibility of the method was found to be excellent as confirmed by the CVs of the within and between assays, which were less than 2.8% and 4.7%, respectively. The recovery rates of the within and between assays were also acceptable at 93.7–101.1% and 96.1–108.5%, verifying the excellent precision of our method.

## 3. Materials and Methods

### 3.1. Chemicals and Reagents

All synthetic DNA oligomers were purchased from Bioneer (Daejeon, Korea). CircLigase ssDNA ligase (100 U/μL) was purchased from Epicentre Biotechnologies (Madison, WI, USA). Phi29 DNA polymerase (10 U/μL) was purchased from Thermo Fisher Scientific, Inc. (Waltham, MA, USA). Exonuclease I and III were purchased from New England Biolabs (Ipswich, MA, USA). SA-AuNP solution was purchased from Cytodiagnostics (Burlington, ON, Canada), and 15% polyacrylamide gels were purchased from Bio-Rad (Hercules, CA, USA). Oligo Clean-Up and Concentration Kit were purchased from Norgen Biotek (Thorold, ON, Canada), and 0.1 M phosphate buffer solution (PB, pH 7.5) and all metallic salts were purchased from Sigma-Aldrich (St. Louis, MO, USA). The nitrocellulose (NC) membrane (CNPF-SN12, width 25 mm, pore size 10 μm) was purchased from MDI Membrane Technologies (Ambala, India). The cellulose acetate (CA) syringe filters (BSS20-CA13, diameter 13 mm, pore size 0.2 μm) were purchased from BioFact (Daejeon, Korea). SYBR Green I and II were purchased from Life Technologies (Eugene, OR, USA). Ultrapure water was purchased from WelGENE (Daegu, Korea). 10× tris-borate-EDTA (TBE) buffer solution and 10× phosphate buffer solution were purchased from Dongin Biotech (Seoul, Korea).

### 3.2. Preparations for Circular Template DNA

For the synthesis of circular template ssDNA, 5 μM of linear 54 mer ssDNA (5′-phosphate-GTCCTCAGTCCCAATAGAAGCGGAGCTTCAAAAAAAAAAAAACGTCTGAAGAGG) in 100 μL of a reaction mixture solution with final 1× reaction buffer, 400 units of CircLigase, 50 μM of ATP, and 2.5 mM of MnCl_2_ was subjected to ligation at 60 °C for 6 h and inactivated at 80 °C for 10 min. Then, to remove residual linear ssDNA, 120 units of exonuclease I and 20 units of exonuclease III were added to the mixture solution and incubated at 37 °C for 4 h and at 80 °C for 20 min, sequentially. The resulting products were purified using the Oligo Clean-Up and Concentration Kit and confirmed by 15% denaturing urea-polyacrylamide gel electrophoresis with 1× TBE buffer at 100 V for 75 min, followed by gel staining with 1× SYBR Green II for 15 min (Figure S1, Supplementary Materials).

### 3.3. Preparations for DNA-Modified AuNPs

An SA-AuNP solution (300 μL, OD_520nm_ = 10) was mixed with 200 μL of 100 μM biotinylated ssDNA probe (5′-AGCGGAGCTTCA-C6SP-biotin). Next, the mixture was incubated at room temperature (18–23 °C) for 3 h with gentle shaking and further incubated at 4 °C overnight. To obtain purified AuNP probes, the solution was washed two times via centrifugation at 6000× *g* for 30 min and resuspended in 1× phosphate buffer at pH 7.5. Two washing steps were then performed using distilled water. The morphologies of AuNP probes were examined by TEM (JEM1010, JEOL, Tokyo, Japan), and additional properties were determined by DLS and zeta potential measurement (Nano ZS90 zetasizer, Malvern Instruments Corp., Worcestershire, UK).

### 3.4. RCA Reaction Test

The fluorescence intensity of SYBR Green II was measured after the RCA reaction to confirm the quantity of ssDNA coils according to the amount of circular template–(T)12 complex. First, different concentrations of circular template–(T)12 complex (0, 0.375, 0.75, 1.5, 3 and 6 μM) in 80 μL of total reaction mixture with final 1× buffer, 75 units of phi29 DNA polymerase, and 0.94 mM of dNTPs (deoxyribonucleotide triphosphates) was reacted at 30 °C for 90 min and inactivated at 65 °C for 10 min. Next, 1 μL of 100× SYBR Green II was added, and the fluorescence intensity was measured using a multi-mode microplate reader (SpectraMax i3x, Molecular Devices, Sunnyvale, CA, USA) after incubation at room temperature for 30 min.

### 3.5. Selectivity for Hg^2+^

To assess the selectivity of the proposed method for mercuric ions, aqueous solutions spiked with 16 different metal ions (Li^+^, Mn^2+^, Ag^+^, Pb^2+^, Co.^2+^, Ba^2+^, Ca^2+^, K^+^, Ni^2+^, Cd^2+^, Zn^2+^, Fe^2+^, Mg^2+^, Na^+^, Cu^2+^, and Hg^2+^) were tested. First, to prepare primer-metal ion mixture, 5 μL of 200 nM (T)12 primer solution was mixed with 5 μL of 16 μM each metal ion solution and subsequently incubated at room temperature for 30 min. Next, 40 μL of RCA reaction mixture was prepared with final 1× reaction buffer, 0.87 mM of dNTPs, 25 units of phi29 DNA polymerase, and 18.75 nM of the circular template. Then, the primer-metal ion mixture was added to the prepared RCA reaction mixture, and RCA reaction was started after applying 30 μL of AuNP probe (OD_520nm_ = 3.3). The RCA reaction was maintained at 30 °C for 30 min and inactivated at 65 °C for 10 min, sequentially. Then, 10 μL of each reaction solution was drop-dried on the NC membrane for 20 min. The results were analyzed using a portable spectrophotometer (RM200QC, X-Rite Co., Neu-Isenburg, Germany). The spectrophotometer uses CIE L*a*b* (CIELAB), which is a color space specified by the CIE (Commission Internationale del’Eclairage). CIELAB described all colors visible to the human eye and was created to serve as a device-independent model to be used as a reference. When a color is expressed in CIELAB, L* defines lightness, a* denotes the red/green value, and b* denotes the yellow/blue value. The value of Δ*E* represents the total color difference, as calculated by the following equation:

Δ*E* = [(ΔL*)^2^ + (Δa*)^2^ + (Δb*)^2^]^1/2^

The selectivity of (T)12 primer toward mercuric ions was also investigated (Figure S3, Supplementary Materials). First, 1 μL of each 500 μM ion solution (Li^+^, Mn^2+^, Ag^+^, Pb^2+^, Co.^2+^, Ba^2+^, Ca^2+^, K^+^, Ni^2+^, Cd^2+^, Zn^2+^, Fe^2+^, Mg^2+^, Na^+^, Cu^2+^, and Hg^2+^) was mixed with 1 μL of 100 μM primer solution and 98 μL of distilled water in each well of a 96-well plate and incubated at room temperature for 1 h. Then, 1 μL of 100× SYBR Green I was added to each well, and the fluorescence intensity was measured using a multi-mode microplate reader (SpectraMax i3x, Molecular Devices, Sunnyvale, CA, USA) after incubation at room temperature for 30 min. A mixed sample containing all metal ions and a control sample without metal ions were also tested under the same conditions.

### 3.6. Quantification of Hg^2+^ by Radial Flow Assay

For quantification of Hg^2+^ using the proposed method, 5 μL of 2 μM (T)12 primer solution was mixed with 5 μL of Hg^2+^ solution at various concentrations (from 0 to 39 μM) and subsequently incubated at room temperature for 30 min. Next, 25 μL of RCA reaction mixture was prepared with final 1× reaction buffer, 0.92 mM of dNTPs, 25 units of phi29 DNA polymerase and 230 nM of circular template. Then, the primer-Hg^2+^ mixture was added to the prepared RCA reaction mixture, and RCA reaction was started after applying 30 μL of AuNP probe (OD_520nm_ = 4). The RCA reaction was maintained at 30 °C for 30 min and inactivated at 65 °C for 10 min, sequentially. Next, 20 μL of reaction solution was drop-dried on the NC membrane for 20 min. The results were analyzed using a portable spectrophotometer. Additionally, distilled water and tap water samples spiked with different concentrations of Hg^2+^ (0, 50, 250, 500, 1000, and 1500 nM) were tested under the same conditions. Tap water was obtained from the laboratory at our institute and filtered through a cellulose acetate syringe filter with a pore size of 0.2 μm before being used for the assay. A surface image of the NC membrane was obtained using SEM (JSM-6380, JEOL, Tokyo, Japan). To investigate the DNA coil–AuNP complexes by AFM, 30 μL of sample solution from the RCA reaction was deposited on a freshly cleaved mica disc and washed with 3 mL of distilled water, followed by drying with a mild stream of N_2_ gas. The morphologies of DNA coil-AuNP complexes were then examined using AFM (XE-70, Park Systems, Suwon, Korea) in tapping mode with noncontact cantilever probes (PPP-NCHR, Park Systems) at a scan rate of 0.3 Hz.

## 4. Conclusions

A novel colorimetric radial flow assay for detection of Hg^2+^ was developed through the use of RCA and probe-modified AuNPs. NC-based paper and a portable spectrophotometer were used for simple and easy analysis of colorimetric results without the need for bulky and expensive equipment or skilled personnel. Through this study, we confirmed that the color intensity change of the inner spot generated by drop-drying of the reaction solution on a paper was selective for Hg^2+^ among metal ions based on the coordinated binding of Hg^2+^ between two thymines. Moreover, great linearity toward Hg^2+^ in tap water samples was obtained with a limit of detection of 22.4 nM. Additionally, the assay displays high precision and reproducibility and demonstrates its utility for Hg^2+^ quantification in real samples. By adopting the analytical method based on radial flow on bare paper and employing a portable spectrophotometer that is not affected by variable lighting conditions or personal skills, the assay provides simple, convenient, and cost-effective detection of Hg^2+^. Additionally, the proposed radial flow assay is a new strategy applied for the first time to on-site detection of Hg^2+^. Furthermore, it has potential to apply to detect not only mercuric ions but also other heavy metal ions by utilizing a target-specific DNA probe acting as a primer for RCA and circular DNA complementary with the primer. On the basis of the results described above, we strongly expect that this method has great potential to be an on-site sensor system for Hg^2+^ under resource-constrained conditions.

## Figures and Tables

**Table 1:** Determination of Hg^2+^ in tap water samples using the proposed radial flow assay.

Samples No.	Added (nM)	Within-Assay			Between-Assay		
		Found (nM) (Mean ^a^ ± SD)	CV ^b^ (%)	Recovery (%)	Found (nM) (Mean ^a^ ± SD)	CV ^b^ (%)	Recovery (%)
1	150	140.5 ± 0.32	1.9	93.7	162.7 ± 0.19	1.2	108.5
2	450	454.8 ± 0.23	1.6	101.1	432.5 ± 0.5	3.3	96.1
3	700	705.6 ± 0.38	2.8	100.8	696.0 ± 0.64	4.7	99.4

^a^ Mean of five determinations. ^b^ % CV, coefficient of variation = 100 × (SD value/mean value).

## Supplementary Materials

The following are available online at http://www.mdpi.com/2079-4991/8/2/81/s1, Figure S1: Urea-PAGE analysis of four serial samples obtained through the formation of circular ssDNA using CircLigase and exonucleases, Figure S2: (A) Dynamic light scattering to determine size distributions of streptavidin-conjugated AuNPs (SA-AuNPs) and biotinylated oligonucleotide-functionalized AuNP (AuNP probes). (B) Zeta-potential data to measure surface charges of SA-AuNPs and AuNP probes. After the introduction of a negatively charged biotinylated oligonucleotide onto the surface of SA-AuNPs, the size and surface charge of the Au probe were altered, Figure S3: Selectivity test of (T)12 primers toward mercury ions. Sixteen different metal ions were used in the experiment, Figure S4: Digital scanned images of the paper after drop-drying of the reaction samples containing different concentrations of Hg^2+^ (0, 50, 250, 500, 1000, and 1500 nM) in: distilled water (A); and tap water (B), Figure S5: (A) Comparison of fluorescence intensities between the control and (T)12 primers after binding with Hg^2+^; and (B) Digital scanned image of the paper after drop-drying of control or (T)12 primer-applied RCA reaction samples, Table S1: Comparison of the proposed radial flow assay with ICP-MS to quantify Hg^2+^, Table S2: Sequences of the control and original primers, and circular templates which are complementary with each primer used for RCA reactions.
