# Supplementary material for: Radial Flow Assay Using Gold Nanoparticles and Rolling Circle Amplification to Detect Mercuric Ions
Source: Nanomaterials (Basel). 2018 Feb 1;8(2):81. doi: 10.3390/nano8020081 (PMC5853713; doi:10.3390/nano8020081)
Supplement: Supplementary file 1 [file nanomaterials-08-00081-s001.pdf]

< Supplementary Materials>

Radial Flow Assay Using Gold Nanoparticles and Rolling Circle Amplification to Detect Mercuric Ions

Tai-Yong Kim [1,2], Min-Cheol Lim [1], Min-Ah Woo [1,*] and Bong-Hyun Jun [2,*]

[1] Food Safety Research Group, Korea Food Research Institute (KFRI), Jeollabuk-do 565-851, Korea; kim.tai-yong@kfri.re.kr (T.-Y.K.); mclim@kfri.re.kr (M.-C.L.)

[2] Department of Bioscience and Biotechnology, Konkuk University, Seoul 143-701, Korea

* Correspondence: mawoo@kfri.re.kr (M.-A.W.); bjun@konkuk.ac.kr (B.-H.J.); Tel.: +82-63-219-9374 (M.-A.W.); +82-2-450-0521 (B.-H.J.)

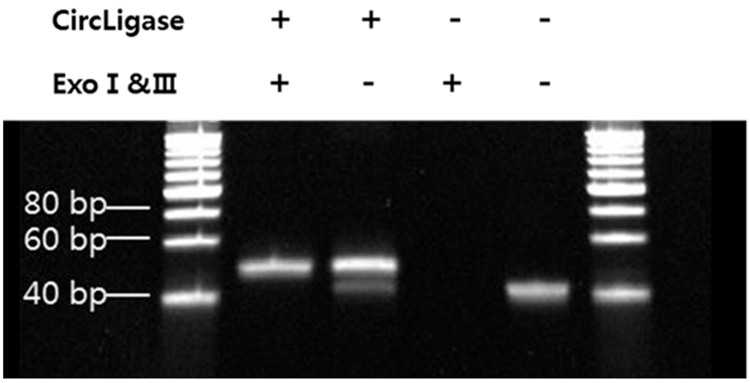

Figure S1. Urea-PAGE analysis of four serial samples obtained through the formation of circular ssDNA using CircLigase and exonucleases. For the synthesis of circular template ssDNA, linear ssDNA with a length of 54 mer was first subjected to ligation using CircLigase. Then, to remove residual linear ssDNA, exonuclease I (Exo I) and exonuclease III (Exo III) were applied to the ligation reaction mixture. The resulting four samples were loaded into the gel and stained with 1× SYBR Green II. As a result, the circularized ssDNA product migrates slower than linear ssDNA and is seen as a band above the linear ssDNA.

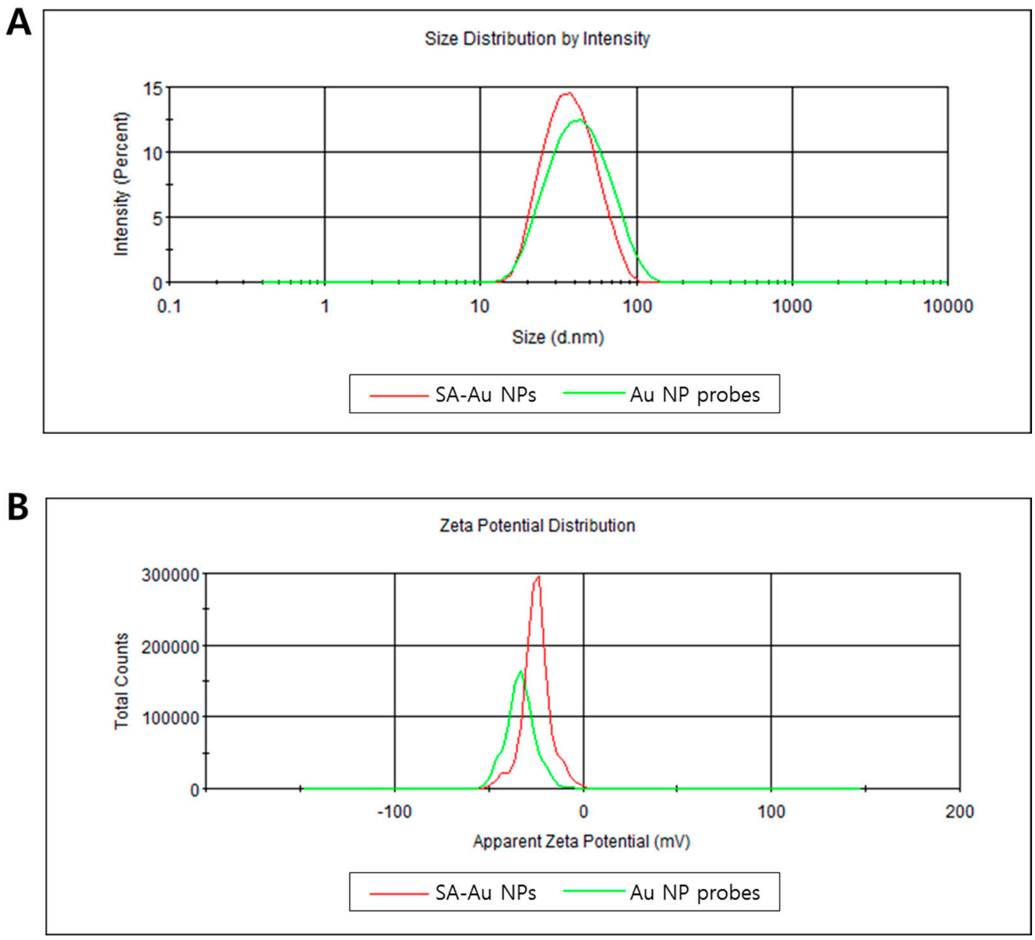

Figure S2. (**A**) Dynamic light scattering to determine size distributions of streptavidin-conjugated AuNPs (SA-AuNPs) and biotinylated oligonucleotide-functionalized AuNP (AuNP probes). The mean diameter of the particles after the introduction of biotinylated oligonucleotides to SA-AuNPs increased from 33.21 to 35.29 nm; (**B**) Zeta-potential data to measure surface charges of SA-AuNPs and AuNP probes. After the introduction of a negatively charged biotinylated oligonucleotide onto the surface of SA-AuNPs, the size and surface charge of the Au probe were altered. In addition, the negative charge became stronger, increasing to -33.8 mV from -24.8 mV. These results show that the oligonucleotides were successfully bound to the surfaces of the SA-AuNPs and were made more stable by nonspecific aggregation through an increased negative surface charge.

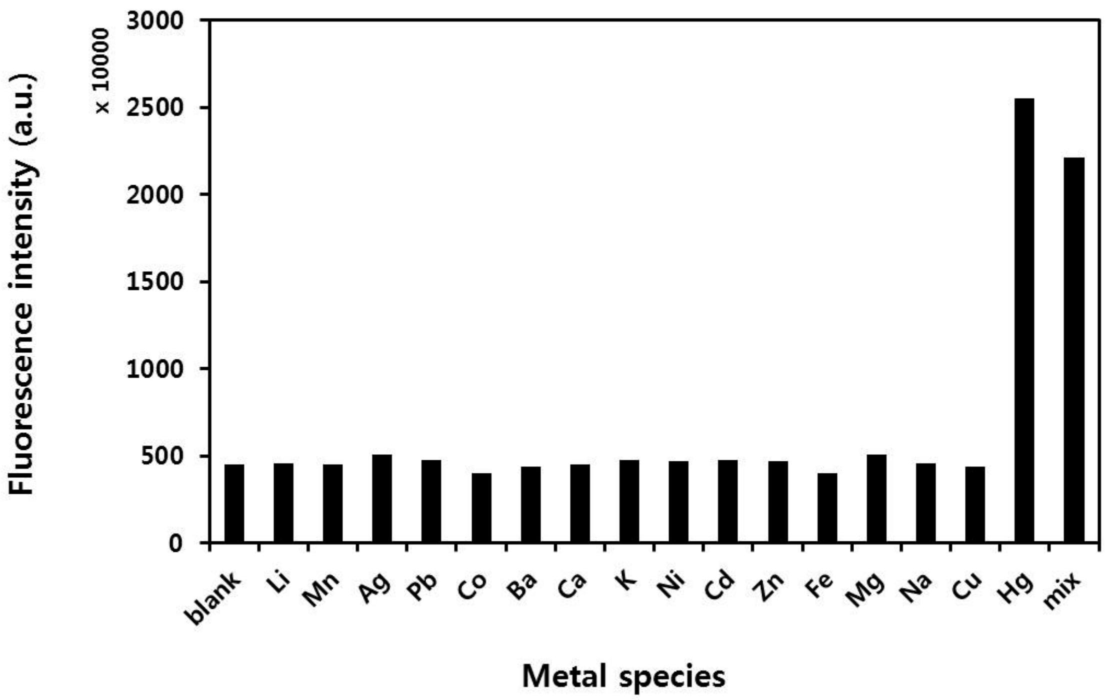

Figure S3. Selectivity test of (T)12 primers toward mercury ions. 16 different metal ions were used for the experiment. The final concentration of each metal ion was 5 μM. Each metal ion solution was mixed with (T)12 primer solution at a final concentration of 1 μM and the final mixture solution was stained with 1 × SYBR Green I. As a result, the high fluorescence intensity of SYBR Green I was observed only in the two samples containing Hg^{2+}. This result shows that the single-stranded primer is converted into a double-stranded complex due to coordinate binding of Hg^{2+} between two thymines.

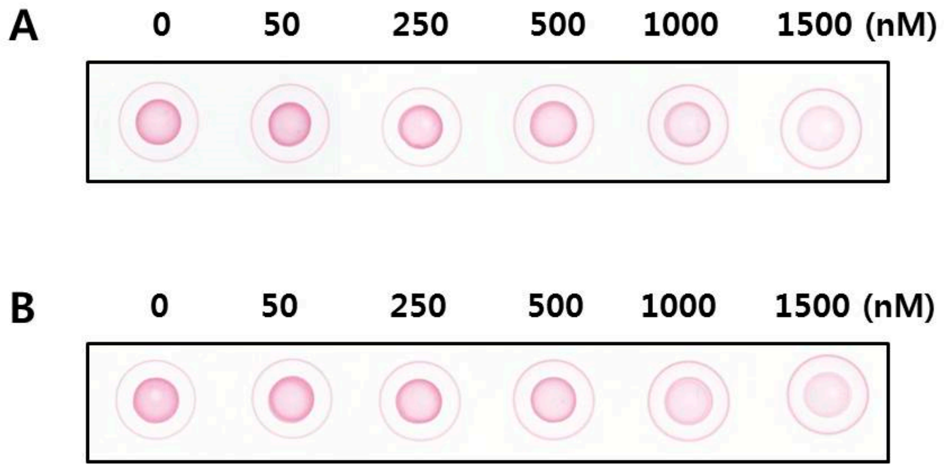

Figure S4. Digital scanned images of the paper after drop-drying of the reaction samples containing different concentrations of Hg^{2+} (0, 50, 250, 500, 1000, and 1500 nM) in distilled water (**A**) and tap water (**B**). In both cases, as the Hg^{2+} concentration increased, the red color spot at the center of the drop gradually faded.

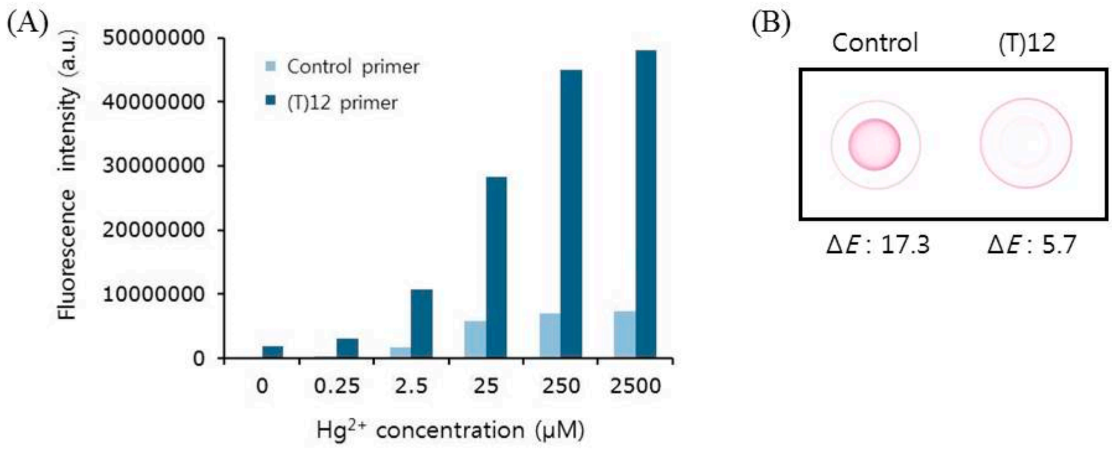

Figure S5. (**A**) Comparison of fluorescence intensities between the control and (T)12 primers after binding with Hg^{2+}; (**B**) Digital scanned image of the paper after drop-drying of control or (T)12 primer-applied RCA reaction samples. Color intensities (ΔE) of the drops were measured by a portable spectrophotometer. The sequences of the control primer and circular template complementary with the primer are shown in Table S2. The experiments were conducted to confirm that our assay results differed from another primer which is not specific for Hg^{2+}-due to the absence of T-T mismatch sites unlike poly T primer. First, 3 μM of Hg^{2+} in each solution containing control or (T)12 primer was incubated at room temperature for 30 min, and the fluorescent signals were measured after treatment of SYBR green I dye which stains double-stranded DNA into each solution. As a result, (T)12 primer showed higher fluorescence intensities than control primer demonstrating that Hg^{2+} coordinated with thymines to form T-Hg^{2+}-T complexes (A). Also, in order to compare the colorimetric results between using the control and (T)12 primer applied to the proposed assay, 65 μL of the final RCA reaction mixture with the same compositions used for the quantitative experiment of this work was prepared under 3 μM of Hg^{2+} condition. The prepared RCA reaction mixture was incubated at 30 °C for 30 min and at 65 °C for 10 min, sequentially. Next, 20 μL of the reaction solution was drop-dried on the NC membrane. As a result, we found that the ΔE value of the drop for (T)12 primer test was lower than that of the drop for control primer test because (T)12 primers inhibited RCA reaction (B).

Table S1. Comparison of the proposed radial flow assay with ICP-MS to quantify Hg^{2+}.

**Samples No.**	**Added (μg/L)**	**ICP-MS method**			**Proposed method**		
		**Found (μg/L) (mean ± SD)**	**CV [a] (%)**	**Recovery (%)**	**Found (μg/L) (mean ± SD)**	**CV [a] (%)**	**Recovery (%)**
1	23.1	19 ± 0.42	5.7	82.3	29.8 ± 0.58	3.2	129
2	285.4	334.3 ± 2.46	6.5	117.1	286.4 ± 0.12	0.7	100.4
3	399.5	375.2 ± 1.38	3.3	93.9	418.9 ± 0.27	1.5	104.9
4	600	552.2 ± 1.68	2.7	92	576.2 ± 0.44	2.6	96

[a] % CV, coefficient of variation = 100 × (SD value/mean value).

* Mean and SD values were obtained from within assay (n = 5).

Table S2. Sequences of the control and original primers, and circular templates which are complementary with each primer used for RCA reactions.

Name.	Control sequences	Original sequences
Primer	5′-AACTCGGAGAAC-3′	5′-TTTTTTTTTTTT-3′
Circular template	5'-phosphate-GTCCTCAGTCCCAATAGAAGCGGAGCTTCAGTTCTCCGAGTTCGTCTGAAGAGG-3′	5'-phosphate-GTCCTCAGTCCCAATAGAAGCGGAGCTTCAAAAAAAAAAAAACGTCTGAAGAGG-3′
